# The impact of professional characteristics and person-centred care on general practitioners’ stress levels. Findings from the cross-sectional PACE GP/FP study in 24 European countries

**DOI:** 10.1080/13814788.2026.2652678

**Published:** 2026-04-14

**Authors:** Katarzyna Nessler, Krzysztof Studzinski, Zalika Klemenc-Ketiš, Heather L. Rogers, Torunn Bjerve Eide, Sara Ares Blanco, Heidrun Lingner, Sanda Kreitmayer, Jesús González-Lama, Esther Van Poel, Didem Kafadar, Kathryn Hoffmann, Thomas Frese, Radost Assenova, Mehmet Ungan, Gorka Vuletić, Erika Zelko, Rosa Megallón-Botaya, Marion Tomičić, Fatima Mendez Lopez, Zoi Tsimtsiou, Miguel J. Mora, Vinita Mahtani, Ewa Wójtowicz, Adam Windak, Goranka Petriček

**Affiliations:** ^a^ Department of Family Medicine, Jagiellonian University Medical College, Kraków, Poland; ^b^ The College of Family Physicians in Poland, Warsaw, Poland; ^c^ Ljubljana Community Health Centre, Ljubljana, Slovenia; ^d^ Department of Family Medicine, Medical Faculty, University of Maribor, Maribor, Slovenia; ^e^ Department of Family Medicine, Medical Faculty, University of Ljubljana, Ljubljana, Slovenia; ^f^ Biocruces Bizkaia Health Research Institute, Bizkaia, Barakaldo, Spain; ^g^ Ikerbasque Basque Foundation for Science, Bilbao, Spain; ^h^ Department of General Practice, Institute of Health and Society, University of Oslo, Oslo, Norway; ^i^ Federica Montseny Health Centre, Gerencia Asistencial Atención Primaria, Servicio Madrileño de Salud, Madrid, Spain; ^j^ Instituto de Investigación Sanitaria Gregorio Marañón, Madrid, Spain; ^k^ Department of Medical Psychologie, Center for Public Health and Healthcare, Hannover Medical School, Hannover, Germany; ^l^ Department for General and Family Medicine, Public Health Center “Dr M. Sehovic” Tuzla, Tuzla, Bosnia and Herzegovina; ^m^ Medical Faculty, University of Tuzla, Tuzla, Bosnia and Herzegovina; ^n^ Maimonides Institute for Biomedical Research of Córdoba (IMIBIC), Reina Sofía University Hospital, Córdoba University, Córdoba, Spain; ^o^ Matrona Antonia Mesa Fernández’ Health Center, Cabra Clinical Management Unit, AGS South of Córdoba, Cabra (Córdoba), Spain; ^p^ Department of Public Health and Primary Care, Ghent University, Ghent, Belgium; ^q^ Cerrahpasa Faculty of Medicine, Department of Family Medicine, Istanbul University-Cerrahpasa, Fatih/İstanbul, Türkiye; ^r^ Department of Primary Care Medicine, Center for Public Health, Medical University of Vienna, Vienna, Austria; ^s^ Institute of General Practice, Faculty of Medicine, University of Halle-Wittenberg, Halle/Saale, Germany; ^t^ Department Urology and General Practice, Faculty of Medicine, Medical University of Plovdiv, Plovdiv, Bulgaria; ^u^ Department of Family Medicine, Ankara University School of Medicine, Ankara, Turkey; ^v^ Department of Psychology, Faculty of Humanities and Social Sciences, University J. J. Strossmayer in Osijek, Osijek, Croatia; ^w^ Faculty of Medicine, Institut of General Medicine, JKU Linz, Linz, Austria; ^x^ Aragones Group of Research in Primary Health Care (GAIAP), Institute for Health Research Aragon (IISAragon), Zaragoza, Spain; ^y^ Department of Medicine, Psychiatry and Dermatology, Faculty of Medicine, University of Zaragoza, Zaragoza, Spain; ^z^ Department of Family Medicine, School of Medicine, University of Split, Split, Croatia; ^aa^ Split-Dalmatia Health Center, Split, Croatia; ^bb^ Department of Physiatry and Nursing, University of Zaragoza, Zaragoza, Spain; ^cc^ Department of Hygiene, Social-Preventive Medicine and Medical Statistics, School of Medicine, Aristotle University of Thessaloniki, Thessaloniki, Greece; ^dd^ Cueva Torres Health Center, Gerencia de Atención Primaria de Gran Canaria, Servicio Canario de Salud, Madrid, Spain; ^ee^ Research Unit University Hospital Nuestra Señora de la Candelaria and Primary Care. Canary Islands Health Care Services, Santa Cruz de Tenerife, Spain; ^ff^ Red de Investigación en Cronicidad, Atención Primaria y Promoción de la Salud-RICAPPS-ISCIII, Spain; ^gg^ Department of Family Medicine, School of Medicine, University of Zagreb, Zagreb, Croatia; ^hh^ Health Centre Zagreb-West, Zagreb, Croatia

**Keywords:** Primary care, general practice, perceived stress, person-centred care, PACE GP/FP study

## Abstract

**Background:**

General practitioners (GPs) face numerous challenges that can contribute to stress. Understanding these factors is crucial for developing interventions to support physician wellbeing and maintain high-quality care.

**Objectives:**

The study aims to explore the factors associated with perceived stress among European GPs, including attitudes towards person-centred care (PCC), demographics, and professional characteristics.

**Methods:**

The PACE GP/FP study is an online, cross-sectional, multi-centred survey conducted in 24 European countries between November 2022 and January 2024. The survey tool included the Perceived Stress Scale (PSS), the Patient-Practitioner Orientation Scale (PPOS), and questions on GPs’ demographics and practice characteristics. Linear mixed models analysed the relationship between these variables and perceived stress.

**Results:**

In total, 3522 GPs were included in the analysis. The mean PSS score indicated moderate levels of stress. Female gender and younger age were associated with increased stress. Also, a higher number of daily patient contacts and a greater perceived responsibility for vulnerable patient populations (e.g. migrants, those with limited social support, or psychiatric vulnerabilities) were significantly associated with higher stress. A stronger patient-centred orientation was associated with lower perceived stress.

**Conclusion:**

The findings have implications for interventions to reduce GP stress, such as training programs promoting PCC, optimising patient contact rates, and providing targeted support for GPs caring for vulnerable patients. Further research is needed to explore these factors’ complex interplay and impact on GP wellbeing.

## Introduction

Healthcare professionals, especially general practitioners (GPs), are significantly more likely to experience work-related stress [1–3]. Chronic work-related stress is associated with adverse outcomes, including burnout, depressive symptoms, and compromised work quality, which may impact patient safety [4,5].

Extensive literature documents the effects of work-related stress on GPs’ mental health, highlighting consequences for patient care quality, an increase in adverse events in patient care, and strained patient-physician relationships [6,7]. Furthermore, personal experiences of work dissatisfaction and limitations may discourage medical students from choosing general practice as their future careers or lead GPs to abandon the specialty, a pressing issue given current GP shortages in many countries [8,9]. However, the relationship between GPs’ working conditions and perceived stress is less thoroughly examined [10,11].

Person-centred care (PCC) has been recognised as a core value and one of the principles of general practice/family medicine by the European branch of the World Organisation of Family Doctors (WONCA Europe) [12]. It adopts a holistic view of patient wellbeing and emphasises the centrality of individual preferences, values, needs, and experiences in healthcare decision-making and treatment planning [13]. PCC positively impacts various aspects of care: patients’ emotional state, satisfaction, and adherence to medical recommendations. It reduces the number of adverse outcomes and patient complaints and improves physician satisfaction and the quality of the consultation [14,15]. Physicians encouraging patient involvement in treatment decisions receive higher ratings for interpersonal manners and technical skills and improve interpersonal dynamics and care outcomes [16].

According to a Delphi expert study of European GPs, trying to be a person-centred doctor is one of eight highly ranked factors associated with work satisfaction [17].

Till now, we have little knowledge of how PCC may impact GPs’ perceptions of work-related stress. Understanding these links is critical to strengthening primary care [18]. Many countries have reorganised healthcare structures and expanded GPs’ roles to increase efficiency and manage costs. These changes, however, may inadvertently contribute to GPs’ stress [19].

This paper aims to answer the following research questions:

1. Is there a relationship between the demographic and professional characteristics of GPs and their perceptions of work-related stress?
2. Is there a relationship between PCC and perceived stress?

## Materials and methods

### Study design

This cross-sectional study was based on self-reported data gathered through an online survey of GPs across 24 European countries as part of the PACE GP/FP study [20]. The PACE GP/FP study was conducted by the Person-Centred Primary Care group in collaboration with the European Association of Quality and Safety in General Practice/Family Medicine (EQuiP) and the European General Practice Research Network (EGPRN), under a collaboration agreement. It was coordinated by Zagreb University (Croatia).

The final survey was translated from English into the primary spoken languages using a forward-backwards translation method. The REDCap application was used [21].

### Study tool

A team of experts, comprising EQUIP and EGPRN primary health care representatives, developed the first and fourth parts of the survey in a collaborative effort. Questions were subject to extensive review and discussions for clarity and relevance across diverse healthcare systems [20]. When responding to the questions, participants were asked to consider their feelings and thoughts related to work. The questionnaire consisted of four parts:

1. Demographic and professional GPs’ practice characteristics (gender, age, years of professional experience, specialist training, practice size, teaching practice, number of GPs in practice, patient population characteristics, payment system
2. 10-item version of the Perceived Stress Scale (PSS).
  The PSS-10 is a validated 10-item tool measuring the degree to which life situations in the past month were perceived as unpredictable, uncontrollable, and overloading. Items are scored on a 5-point Likert scale from 0 (never) to 4 (often), resulting in a total score range of 0 to 40. Higher values indicate more significant perceived stress. It is a standardised instrument with moderate to high reliability (Cronbach’s alpha = 0.78 to 0.91) and confirmed validity. While the Minimal Clinically Important Difference (MCID) can vary by population, research in similar clinical contexts often suggests a change of approximately 2.8 to 3 points as a meaningful threshold for the PSS-10 [22].
3. Patient-Practitioner Orientation Scale (PPOS).
  The PPOS consists of 18 statements assessing whether a respondent prefers a disease-oriented or patient-centred approach. Responses use a 6-point Likert scale (1 = strongly agree to 6 = strongly disagree). Respondents are categorised based on mean scores: Patient-centred: >5.00. Moderately patient-centred: 4.57–5.00 or doctor-centred: <4.57. It measures two subscales: sharing (patient participation in decision-making) and caring (importance of patient emotions and life circumstances). The tool has moderate to high reliability (Cronbach’s alpha = 0.75 to 0.88) and has been extensively validated across various languages and countries, a subject of separate analysis. It assesses the doctor--patient relationship with higher values indicating more agreement with patient-centred (vs. disease-oriented) care during clinical encounters [23].
4. Facilitators and barriers to person-centred care in everyday practice (workload volume, time constraints, number of contacts with patients per day, practice structure and collaboration, organisational culture).

### Sampling and study participants

Data for this study were gathered online between November 2022 and January 2024. The data collection period varied by country, lasting from 10 to 52 weeks. Data collection utilised a convenience sampling approach through diverse recruitment channels, including national professional associations and GP-specific mailing lists. Eligible general practitioners (GPs) were contacted *via* email with a direct link to the online survey. To increase participation, consortium partners in each country were required to issue at least one follow-up reminder. While most participating countries implemented a nationwide sampling strategy, data collection in select nations was limited to specific geographic regions.

The recruitment process followed a structured documentation protocol, with the PACE GP/FP project setting a target of at least 100 GPs per participating country [20]. Each participant received a unique, country-specific survey link. All participants were required to provide written informed consent on the first page of the online survey before proceeding.

### Statistical analysis

Statistical analysis was performed by SPSS software (version 29.0.2.0). Of the 3,813 records eligible for analysis, the primary outcome measure (PSS score) was missing in 220 cases (5.8%), resulting in a baseline of 3,593 records. 3,813 records were eligible for analysis; the primary outcome measure (PSS score) was missing in 220 cases (5.8%), resulting in a baseline of 3,593 records. Seventy-one (2% of 3593) unreliable records were also excluded. The following records were deemed unreliable: physicians who started working as doctors at the age of 22 or younger and/or whose work experience was equal to 0 years, and/or whose total number of patients covered by their practice was less than 100. After applying these exclusion criteria, 3,522 records remained and were included in the final analysis.

Due to the clustering of respondent practices in countries, linear mixed models’ analysis was undertaken with the continuous PSS value as the outcome variable. Independent quantitative variables with outliers, as reflected in standard deviations (number of patients, number of contacts per day), were coded as categorical variables by quartile.

The presence of outliers significantly affected the SD; therefore, to ensure a more robust analysis, in regression models, the variables were divided into quartiles and analysed.

Four models were tested using a stepwise approach with the null model (Model I) permitting the calculation of the intraclass correlation coefficient (ICC), assessing the proportion of the variance in the outcome variable that can be explained by country. In subsequent models, we added GPs’ characteristics (Model II), practice characteristics (Model III), and PSS value (Model IV) as fixed effects. Akaike’s Information Criterion (AIC) and −2 log-likelihood values were used as goodness-of-fit model criteria. The likelihood ratio test was used to compare model fit between the nested models. The boundary values for the criterion of statistical significance (p, two-fold) were determined at *p* < 0.05.

## Results

### Respondent characteristics

3593 GPs from 24 countries participating in the PACE study provided complete responses to questions on perceived work-related stress. Of these, 3522 were eligible to be analysed. 67.5% were female, and 74.2% reported having specialist training. Other features of their demographic and professional characteristics are presented in Table 1.

**Table 1.** Main characteristics of the study participants.

Participants characteristic (*n* = 3522)*	Mean ± SD	Median	First Quartile	Third Quartile
Age (*n* = 3511)	46.5 ± 11.7	46.1	37.0	56.0
Years of professional experience (*n* = 3471)	15.8 ± 11.2	14.0	6.0	23.75
Number of patients under the GP care (*n* = 3473)	3282 ± 7634	1800	1400	2700
Number of contacts with patients per day (*n* = 3504)	47.7 ± 54.4	40.0	30	55
Perceived Stress Scale (PSS) mean value (scale 0–40 points) (*n* = 3522)	17.3 ± 6.5	17.0	13.0	22.0
Patient- Practitioner Orientation Scale (PPOS) mean value (scale 1–6 points) (*n* = 3397)	4.2 ± 0.7	4.2	3.8	4.7

*missing data ranged between 0 and 3,5% for particular variables.

**Figure 1.**
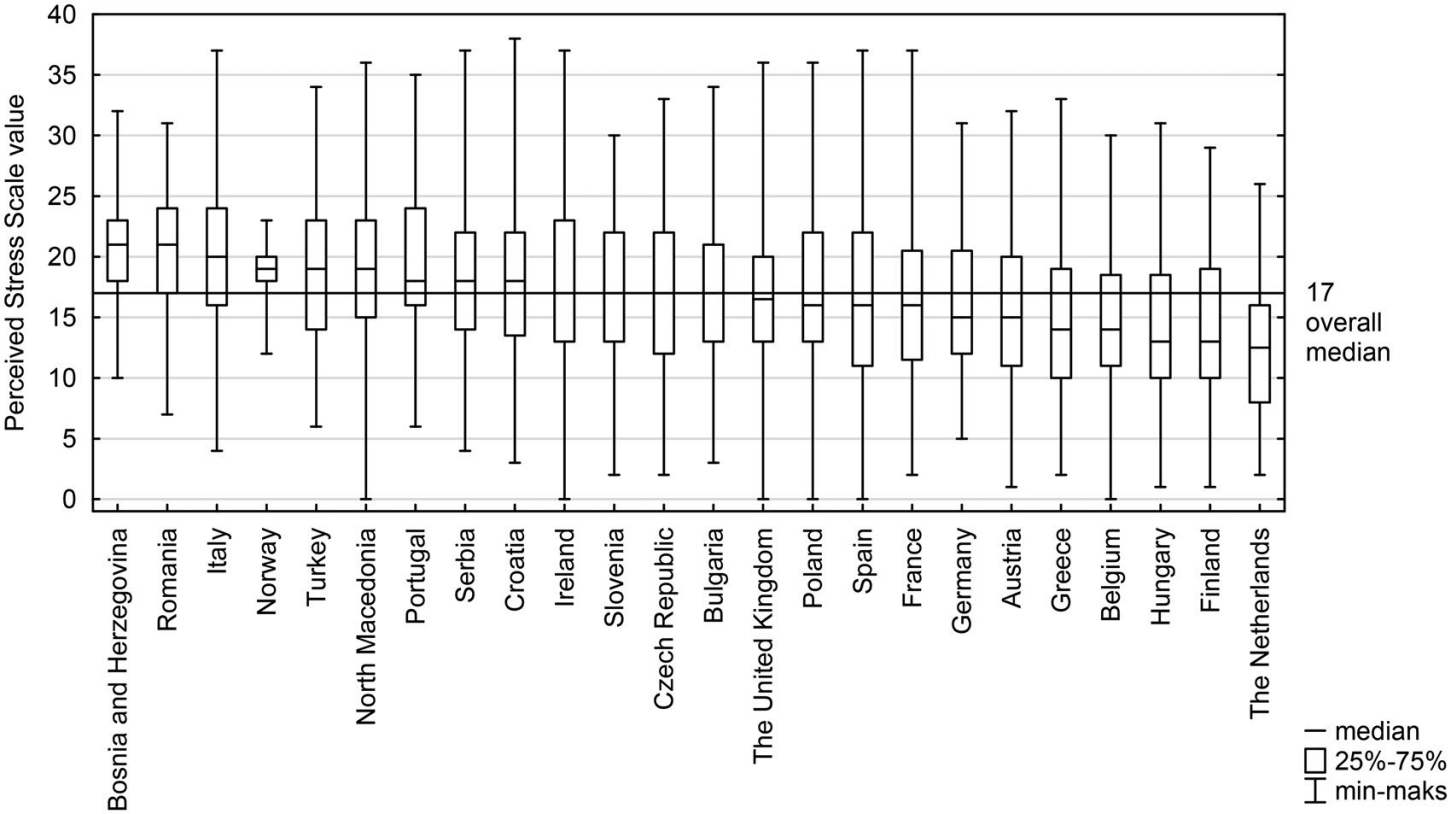
Comparison of PSS scores across countries, presented by value.

The mean PSS value on the 0–40 score scale was 17.5. The highest average stress levels were observed in Bosnia and Herzegovina and Romania, while the lowest were in the Netherlands and Finland. Detailed information on the PSS score values across participating countries is presented in Figure 1.

### Practice characteristics

Table 2 provides information regarding the participating practices and the perception of participants’ responsibility for certain vulnerable patient groups, compared with the average primary care practice in each country. More than half of the practices operated under a capitation payment system (51.1%), approximately one-fourth on a fee-for-service model, while the remainder was based on alternative or mixed payment methods. The majority of the practices served as teaching practices (55.6%). Most practices were larger, with 53.9% employing more than two GPs, while one third (31.1%) were single-GP practices. For both patients with a migration background (facing language barriers) and those with limited health literacy, nearly half of the participants reported their responsibility to be approximately the average (46.1%). Over half of the participants (52.9%) perceived their responsibility for patients with financial problems to be approximately the average, while 21.7% reported a responsibility level above average.

**Table 2.** Characteristics of the practices.

Total	N 3522	%
**Payment system**		
Fee-for-service	849	24.1
Capitation	1798	51.1
Other	875	24.8
**Teaching practice**		
Yes	1958	55.6
No	1564	44.4
**Number of GPs in practice**		
1	1096	31.1
2	526	14.9
>2	1900	53.9
**Number of patients compared to the average primary care practice**		
**- with a migration background and difficulty speaking the local language**		
Above average	567	17.1
Approximately the average	920	46.1
Below average	1672	32.9
I do not know	363	3.9
**- with limited health literacy**		
Above average	602	17.1
Approximately the average	1624	46.1
Below average	1157	32.9
I do not know	139	3.9
**- with financial problems**		
Above average	764	21.7
Approximately the average	1864	52.9
Below average	752	21.4
I do not know	142	4.0
**– with a psychiatric vulnerability**		
Above average	722	20.5
Approximately the average	2126	60.4
Below average	547	15.5
I do not know	127	3.6
**- over the age of 70**		
Above average	1276	36.2
Approximately the average	1748	49.6
Below average	437	12.4
I do not know	61	1.7
**- with chronic conditions**		
Above average	1387	49.4
Approximately the average	1902	54.0
Below average	178	5.1
I do not know	55	1.6
**- with little social support**		
Above average	642	18.2
Approximately the average	1762	50.0
Below average	929	26.4
I do not know	189	5.4
**- women and pre-school children**		
Above average	409	11.6
Approximately the average	1519	43.1
Below average	1379	39.2
I do not know	215	6.1

### GPs’ perceived stress

The linear mixed model analysis of variables associated with GPs’ perceived stress progressed through four models, with Model IV being the most comprehensive. Female GPs reported higher levels of stress (1.51 points more on the PSS scale in the final model) compared to their male counterparts. Increasing age was associated with reduced stress levels (−0.05 points less on the PSS scale per year of life in the final model). GPs with a higher average number of daily patient contacts reported elevated perceived stress levels. Compared to doctors with the lowest number of contacts (up to 30), those with 31–40 contacts scored 0.78 points higher, and those with 41–55 contacts scored 1.36 points higher, and those with the highest number of contacts (more than 55) scored 1.18 points higher in the final model. Similarly, GPs who more frequently treated patients from vulnerable groups experienced higher stress levels (above average number of patients with migration backgrounds: 1.04 points higher, limited social support: 0.97 points higher, psychiatric vulnerabilities: 1.17 points higher). GPs working in practices remunerated on a fee-for-service basis had a marginally lower likelihood of experiencing stress compared to those paid by capitation. Furthermore, GPs with higher PPOS value, indicating a more patient-centred orientation, had a reduced likelihood of experiencing stress (1.42 points lower) compared to those with a more doctor- or disease-centred approach. The study revealed that every 1-point increase in the average PPOS score corresponded to a 1.42-point decrease in the PSS.

Detailed data on all four models included in the linear mixed model analysis are presented in Supplementary file 1.

The analysis revealed that the GP’s age and experience were highly correlated (*r* = 0.89, *p* < 0.001). The final model did not include variables for the physician’s specialty training (found to be not significant) or years of experience. The latter was excluded due to its high correlation with the respondent’s age. The study identified significant intercorrelations between various patient-related stressors. Specifically, the GPs’ subjective assessment of managing patients with financial difficulties was moderately correlated with their assessment of patients lacking social support (*r* = 0.55, *p* < 0.001), those with limited health literacy (*r* = 0.56, *p* < 0.001), and those with psychiatric problems (*r* = 0.40, *p* < 0.001). Furthermore, a perceived increase in patients with psychiatric issues was linked to a higher prevalence of patients with limited health literacy (*r* = 0.36, *p* < 0.001). When entered into the multivariate models, only psychiatric problems remained a significant independent predictor of PSS scores, suggesting that the other social factors may overlap significantly in their impact on doctors’ stress.

## Discussion

### Summary of main findings

The mean Perceived Stress Scale result highlights a moderate level of stress among GPs, consistent with the demands of their role. The Patient-Practitioner Orientation Scale result indicates that GPs generally lean towards a patient-centred approach. A higher Patient-Practitioner Orientation Scale score was associated with lower stress perceived. We observed gender differences in stress levels, with female GPs reporting slightly higher stress than their male counterparts. Age also emerged as a significant factor, with increasing age associated with reduced stress levels.

GPs with a higher average number of daily patient contacts reported elevated stress. Similarly, the GPs’ subjective assessment of being responsible for a higher number of patients from vulnerable groups, such as those with migration backgrounds, limited social support, or psychiatric vulnerabilities, was associated with higher stress levels.

### Comparison with existing literature

Our results indicate that GPs reported a moderate level of stress, slightly higher among female than male practitioners. It has been described that female doctors spend more time with their patients in primary care [24]. This aligns with trends noted in the literature, which consistently highlight gender disparities in stress and coping mechanisms among healthcare providers [25]. Our study showed that increasing age correlates with slightly reduced stress levels, adding evidence to the mixed findings in the literature. Some studies suggest that older GPs develop resilience over time, potentially decreasing perceived stress from daily practices and adjusting their practice to minimise stress. It may also be the result of longer practice, resulting in better knowledge of the problems of their patients. Also, older GPs may have created more effective coping mechanisms for dealing with stress over their careers [26]. Several studies also reported a higher proportion of stress in younger primary care doctors [25,27]. Other studies found that younger GPs experience higher stress levels due to high patient loads and systemic challenges [28]. They may experience higher stress due to the pressures of establishing their practice and meeting performance expectations.

Our observation that a higher average number of daily patient contacts correspond with increased stress reflects concerns raised in prior studies about workload and its direct impact on stress. A higher number of patient contacts likely translate to increased workload, longer hours, and less time for breaks and personal life, leading to burnout.

Treating patients from vulnerable backgrounds has been associated with emotional exhaustion and secondary trauma among healthcare providers [28]. Patients from vulnerable groups often have complex medical and social needs, requiring more time, resources, and emotional support from GPs. Also, addressing these patients’ social determinants of health can be challenging and emotionally demanding for GPs. Moreover, GPs may feel limited in their ability to adequately address the complex needs of vulnerable patients due to resource constraints within the healthcare system [28].

Our findings suggest that financial structures may influence GP stress levels by mediating the relationship between workload and compensation. Systems providing direct financial recognition for increased effort, along with greater scheduling flexibility, appear to offer protective benefits against work-related stress.

The results that higher Patient-Practitioner Orientation Scale values are associated with reduced stress levels support existing literature suggesting that a patient-centred approach can mitigate stress by improving relationships and communication between GPs and patients, leading to a more satisfying practice experience [28,29]. This approach emphasises empathy, communication, and shared decision-making, leading to stronger doctor-patient relationships and increased job satisfaction, potentially reducing stress. Simultaneously, it is also essential to consider that less stress makes GPs more willing and able to practice person-centred care.

### Strengths and limitations

This study has several strengths, including its large sample size and the use of validated questionnaires, which enhance the reliability of the findings. However, some limitations should be acknowledged. The survey’s reliance on self-selected participants introduces volunteer bias that could influence reported findings, though the large sample size helps to minimise this effect. While the lack of documented response rates presents a known limitation for certain analyses, the study prioritised inclusivity and the comprehensive use of all available data from participating countries.

The cross-sectional nature of the data also means we can only identify associations, not direct causal relationships. Furthermore, the voluntary submission of data makes it impossible to acquire precise national population data and calculate country-specific minimum sample sizes, thus preventing us from reporting results at the country level. Although random sampling is unfeasible, our multi-centre approach utilised specific strategies designed to reduce potential biases. Instead of objective patient population data, this study relied on GPs’ estimations of their practice populations and the average practice population nationwide, which may have introduced additional variability. The results are likely influenced by the GPs’ training and system-level factors like the political, economic, and organisational aspects of providing services in each country. Unfortunately, these factors are difficult to quantify.

While our findings reached statistical significance, we acknowledge the distinction between statistical and clinical relevance. The observed reduction in stress was 1.5 points on the PSS-10, which falls below the established Minimal Important Difference (MID) of 2.19–2.66 points [30]. Consequently, we cannot conclude that this change is clinically meaningful at this stage. Despite this, we believe these results represent a significant ‘signal’ in the data that warrants scientific attention. We present these findings with caution—not as a definitive clinical conclusion, but to alert the community to a trend that requires further investigation using larger cohorts and more sensitive longitudinal tools to uncover the underlying mechanisms at play.

According to our knowledge, this is the most extensive study on GPs′ job-related stress, including participants from 24 European countries. It is the first time the Perceived Stress Scale and GPs’ practice characteristics have been assessed in combination across Europe on such a large scale. The sample is quite diverse in its characteristics, encompassing countries with varying levels of gross domestic product, investment in the healthcare system, reimbursement schemes, practices from both rural and urban areas, and a range of GP attributes.

### Implications for research and/or practice

The findings have important implications for healthcare policy and practice. Interventions aimed at reducing GP stress should include training programs. These programs should foster practice environments that encourage a patient-centred orientation, which may help reduce stress. Policies to optimise patient contact rates are essential. Providing additional support for GPs treating vulnerable populations is also necessary. While men and women report similar stress levels, the source of that stress often differs. Since the workforce is predominantly female, the goal should be to sustain a healthy workforce by addressing specific stressors that disproportionately affect women. The time allocated to attend to each patient could influence these results. Future analysis should consider this time allocation to understand its role better. The association between remuneration models and stress warrants further research. Some healthcare systems, incentives geared towards efficiency, and high patient volume may inadvertently contribute to GP stress. The need to meet productivity targets and financial pressures to manage patient populations within fixed budgets can create a challenging environment. Further research is needed to fully understand these factors’ complex interplay and impact on GP stress levels.

## Conclusion

Several demographic and practice-related factors appear to be associated with elevated stress levels among European General Practitioners. These include being female, working at a younger age, managing a high volume of daily patient contacts, and serving populations with a greater concentration of vulnerable patients (such as migrants, individuals with limited social support, or psychiatric issues). A patient-centred approach is associated with lower stress, promoting PCC through training may improve GP wellbeing.

## Supplementary Material

Supplemental Material

## Data Availability

The dataset relied on in this article is available from the corresponding author upon reasonable request.
