# Supplementary material for: The impact of professional characteristics and person-centred care on general practitioners’ stress levels. Findings from the cross-sectional PACE GP/FP study in 24 European countries
Source: Eur J Gen Pract. 2026 Apr 14;32(1):2652678. doi: 10.1080/13814788.2026.2652678 (PMC13081335; doi:10.1080/13814788.2026.2652678)
Supplement: Supplemental Material [file IGEN_A_2652678_SM5850.zip › IGEN_A_2652678_suppl_data/ejgp-2025-0035-File002.docx]

Supplementary File 1 Results of linear mixed model analysis of variables associated with GPs’ perceived stress

|  | Model I | | Model II | | Model III | | Model IV | |
| --- | --- | --- | --- | --- | --- | --- | --- | --- |
|  | Fixed effect estimate (95% CI) | p-value | Fixed effect estimate (95% CI) | p-value | Fixed effect estimate (95% CI) | p-value | Fixed effect estimate (95% CI) | p-value |
| **Intercept** | 17.1  (16.3; 18.0) | <0.001 | 19.22  (17.99; 20.44) | <0.001 | 16.76  (15.19; 18.34) | <0.001 | 22.60  (20.33; 24.86) | <0.001 |
| **Gender** |  |  |  |  |  |  |  |  |
| Male |  |  | Ref. |  | Ref. |  | Ref. |  |
| Female |  |  | 1.15  (0.69; 1.61) | p<0.001 | 1.39  (0.90; 1.89) | p<0.001 | 1.51  (1.01; 2.00) | p<0.001 |
| **Age** |  |  | -0.06  (-0.08; -0.04) | p<0.001 | -0.06  (-0.08; -0.03) | p<0.001 | -0.05  (-0.08; -0.03) | p<0.001 |
| **Average daily patient contacts in practice (quartiles)** |  |  |  |  |  | p<0.001 |  | p=0.003 |
| <=30 |  |  |  |  | Ref. |  | Ref. |  |
| 31-40 |  |  |  |  | 0.74  (0.07; 1.42) | p=0.031 | 0.78  (0.10; 1.46) | p=0.025 |
| 41-55 |  |  |  |  | 1.52  (0.78; 2.26) | p<0.001 | 1.36  (0.61; 2.10) | p<0.001 |
| 56+ |  |  |  |  | 1.29  (0.49; 2.10) | p=0.002 | 1.18  (0.37; 1.99) | p=0.004 |
| **Patient population: migration** |  |  |  |  |  | p=0.011 |  | p=0.005 |
| Below average |  |  |  |  | Ref. |  | Ref. |  |
| Approximately average |  |  |  |  | 0.51  (-0.03; 1.04) | p=0.065 | 0.54  (0.00; 1.08) | p=0.050 |
| Above average |  |  |  |  | 0.97  (0.31; 1.64) | p=0.004 | 1.04  (0.35; 1.74) | p=0.002 |
| **Patient population: little social support** |  |  |  |  |  | p=0.022 |  | p=0.019 |
| Below average |  |  |  |  | Ref. |  | Ref. |  |
| Approximately average |  |  |  |  | 0.68  (0.12; 1.25) | p=0.017 | 0.67 (0.10; 1.23) | p=0.020 |
| Above average |  |  |  |  | 0.92  (0.19; 1.65) | p=0.014 | 0.97 (0.24; 1.71) | p=0.010 |
| **Patient population: perceived psychiatric vulnerability** |  |  |  |  |  | p=0.007 |  | p=0.006 |
| Below average |  |  |  |  | Ref. |  | Ref. |  |
| Approximately average |  |  |  |  | 0.16  (-0.54; 0.85) | p=0.660 | 0.24  (-0.46; 0.94) | p=0.504 |
| Above average |  |  |  |  | 1.08  (0.24; .1.93) | p=0.012 | 1.17  (0.32; 2.02) | p=0.007 |
| **Payment system** |  |  |  |  |  | p=0.042 |  | p=0.022 |
| Capitation |  |  |  |  | Ref. |  | Ref. |  |
| Fee for service |  |  |  |  | -0.07  (-0.75; 0.61) | p=0.833 | -0.04  (-0.72; 0.64) | p=0.901 |
| Other |  |  |  |  | 0.96  (0.15; 1.77) | p=0.020 | 1.07  (0.26; 1.88) | p=0.010 |
| **PPOS** |  |  |  |  |  |  | -1.42  (-1.82; -1.01) | p<0.001 |
| ICC | 0.086 |  | 0.073 |  | 0.087 |  | 0.077 |  |
| Intercept variance (s.e.) | 3.40 (1.15) | p=0.003 | 3.07 (1.05) | p=0.003 | 3.68 (1.24) | p=0.003 | 3.12 (1.08) | p=0.004 |
| Residual variance (s.e.) | 39.32 (0.94) | p<0.001 | 38.68 (0.93), | p<0.001 | 38.33 (1.00) | p<0.001 | 37.06 (1.00) | p<0.001 |
| Model information |  |  |  |  |  |  |  |  |
| Akaike’s Information Criterion (AIC) | 22988.96 |  | 22682.49 |  | 19234.49 |  | 18535.98 |  |
| -2 Log Likelihood | 22984.96 |  | 22678.49 |  | 19230.49 |  | 18531.98 |  |
| Likelihood ratio test |  |  | 306.47 (df=2) | p<0.001 | 3448.00 (df=11) | p<0.001 | 698.51 (df=1) | p<0.001 |
