# Supplementary material for: The impact of professional characteristics and person-centred care on general practitioners’ stress levels. Findings from the cross-sectional PACE GP/FP study in 24 European countries
Source: Eur J Gen Pract. 2026 Apr 14;32(1):2652678. doi: 10.1080/13814788.2026.2652678 (PMC13081335; doi:10.1080/13814788.2026.2652678)
Supplement: Supplemental Material [file IGEN_A_2652678_SM5850.zip › IGEN_A_2652678_suppl_data/ejgp-2025-0035-File003.docx]

Supplementary file 2

Countries presented according to the PSS mean value

Number of respondents per country (total 3522)

| Country | Number of respondents  (N) | PSS Mean value | PSS SD |
| --- | --- | --- | --- |
| Bosnia and Herzegovina | 80 | 20.73 | 4.33 |
| Romania | 94 | 20.00 | 4.70 |
| Italy | 107 | 19.80 | 6.26 |
| Norway | 109 | 18.90 | 2.03 |
| Portugal | 83 | 18.84 | 5.83 |
| Turkey | 123 | 18.80 | 5.81 |
| North Macedonia | 281 | 18.74 | 5.93 |
| Serbia | 360 | 18.25 | 5.74 |
| Ireland | 193 | 17.89 | 7.11 |
| Croatia | 208 | 17.80 | 5.96 |
| Slovenia | 111 | 17.25 | 5.97 |
| Czech Republic | 158 | 17.18 | 6.56 |
| Poland | 95 | 17.12 | 6.60 |
| The United Kingdom | 66 | 16.92 | 6.54 |
| Bulgaria | 121 | 16.70 | 6.29 |
| Spain | 553 | 16.58 | 7.26 |
| France | 104 | 16.41 | 7.36 |
| Germany | 104 | 15.88 | 6.47 |
| Austria | 114 | 15.29 | 6.45 |
| Greece | 113 | 15.10 | 6.75 |
| Belgium | 104 | 14.94 | 6.26 |
| Hungary | 88 | 14.15 | 6.08 |
| Finland | 131 | 13.85 | 5.91 |
| The Netherlands | 22 | 12.73 | 6.17 |
