# Supplementary figures and images for: The impact of professional characteristics and person-centred care on general practitioners’ stress levels. Findings from the cross-sectional PACE GP/FP study in 24 European countries
Source: Eur J Gen Pract. 2026 Apr 14;32(1):2652678. doi: 10.1080/13814788.2026.2652678 (PMC13081335; doi:10.1080/13814788.2026.2652678)

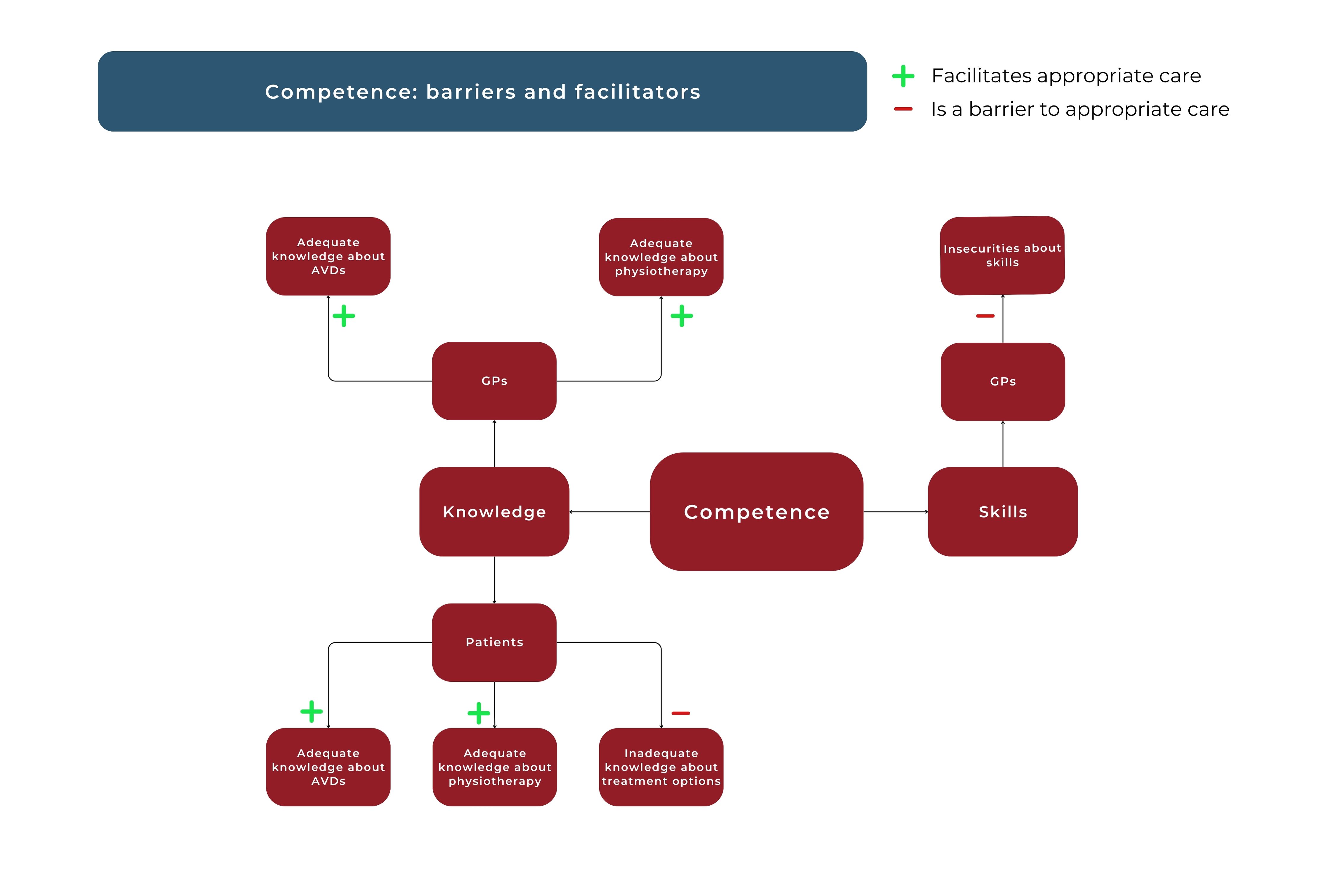

Supplement: Supplemental Material [file IGEN_A_2652678_SM5850.zip › IGEN_A_2652678_suppl_data/ejgp-2025-0201-File004.jpg]

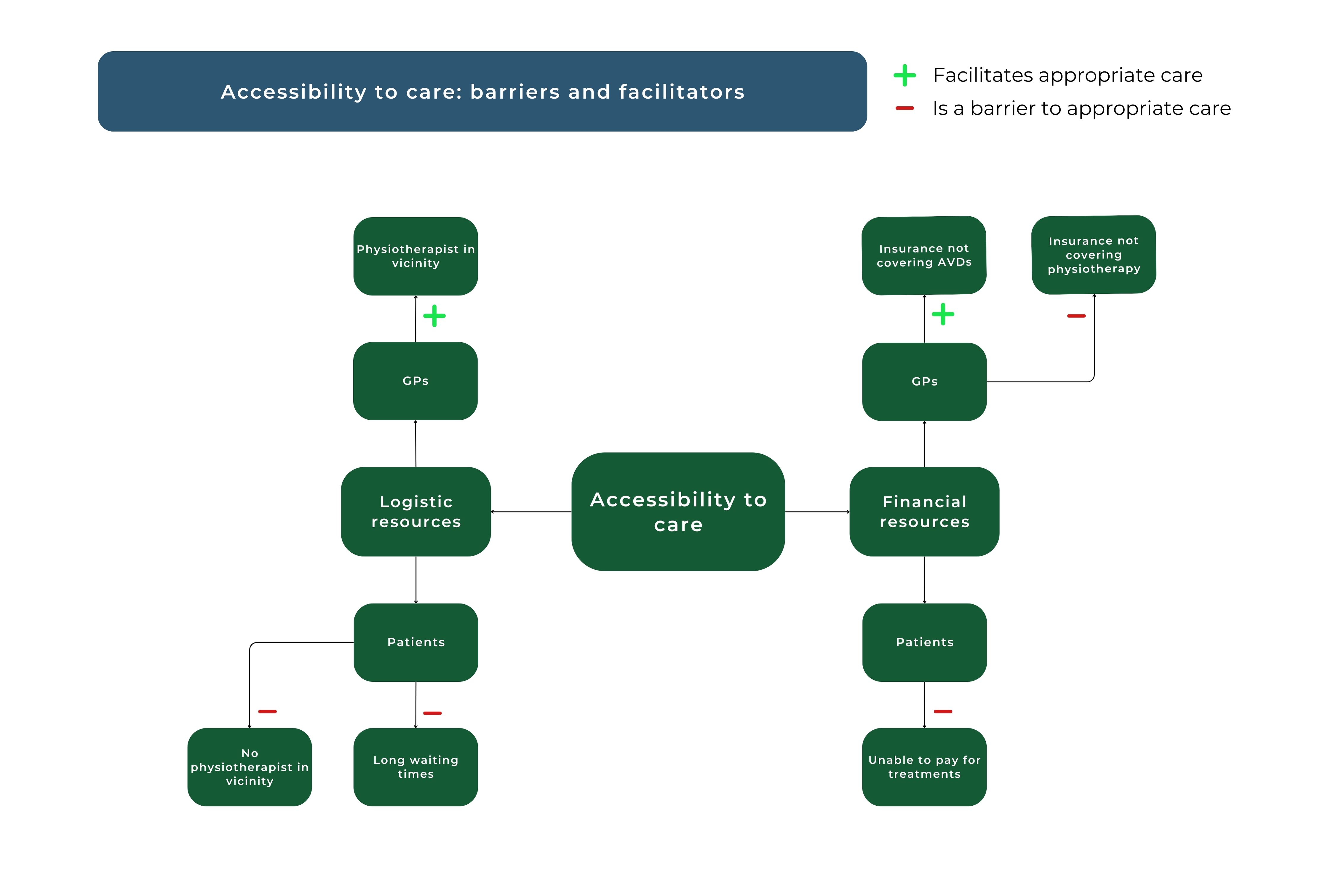

Supplement: Supplemental Material [file IGEN_A_2652678_SM5850.zip › IGEN_A_2652678_suppl_data/ejgp-2025-0201-File007.jpg]
